# Ancestral Wisdom in Cross-Cultural Nursing

**DOI:** 10.1097/AJN.0000000000000254

**Published:** 2026-02-19

**Authors:** Devyn Nixon

**Affiliations:** **Devyn Nixon** is an assistant professor at Western Washington University in Bellingham. Contact author: nixond@wwu.edu. The author has disclosed no potential conflicts of interest, financial or otherwise.

## Abstract

How nurses can engage with the patient experience beyond biomedical instruction

**Figure:**
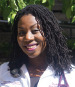
Devyn Nixon

Culture is often defined in health care as language, belief, value systems, and practices shared by a group of people, yet its expression is far more fluid. Culture carries ancestral memory, rituals, foodways, prayer, storytelling, and ways of knowing that may never appear in a medical record. Patients do not enter our care as isolated individuals; they arrive shaped by families, migrations, ecosystems, land, and memory.

In 2022, I created the Health Immemorial Framework, first discussing it on my podcast, The Purple Stethoscope.^1^ Immemorial means existing beyond recorded time—having knowledge older than documentation and textbooks that is often passed down orally through lineage rather than literature. As nurses, honoring ancestral knowing does not replace evidence-based practice. Instead, it strengthens it by acknowledging that health is formed through stories, history, and relationships. As nurses care for increasingly diverse populations, there is a growing imperative to understand how culture, ancestral knowledge, and shared memory inform health behaviors.

**The Health Immemorial Framework**. This framework is a shift in perception rather than a novel intervention. It offers an inclusive, holistic, and transcultural means of connecting with patients to promote health with cultural humility. Rather than centering on the Western ideals of diet, exercise, individualism, and intellectualism, the framework broadens those ideals to include nourishment, movement, community, and awe, which expands the understanding of health-promoting behaviors without dependence on cultural competence.^1^ Below, I break down the shift that could happen if this framework was implemented.

*From diet to nourishment*. Diet is restrictive and prescriptive. Nourishment is expansive. It asks what feeds the body, spirit, and community. Across cultures, nourishment may include soup prepared by an elder, herbs from a garden, food shared during grief, or meals associated with faith practice. Nurses can ask patients, “What foods or practices help you feel strong during illness?” This question validates history and opens clinical dialogue.

*From exercise to movement*. Exercise implies task and measurement. Movement recognizes play, dance, walking to the mailbox, or tending to grandchildren as health promoting. Many cultures do not separate movement from living, as it is woven into daily routine. Nurses may document, “Patient identifies dancing with family as primary form of movement. Encouraged continuation as tolerated.” Care is collaborative, not corrective.

*From individualism to community*. Western health care often elevates independence as the highest ideal. But many cultural traditions view wellness as interdependent. Decision-making may involve elders, faith leaders, neighbors, or kin. In practice, nurses can ask, “Who supports you in health decisions, and how can we include them?” Engaging community aligns treatment with patient reality, improving adherence and emotional safety.

*From intellectualism to awe*. Intellectualism values reasoning and instruction. Awe values wonder, spirituality, and meaning-making. Patients may describe feeling connected to ancestors, land, God, or ritual during healing. Awe supports coping, hope, and grounding during illness. Rather than assessing spirituality as a checkbox, nurses can ask, “What brings you peace when you face health challenges?”

**Application in clinical practice**. The Health Immemorial Framework is not culture-specific; it is culture-grounded. It allows any nurse to care more deeply for any patient, regardless of shared identity. This approach is especially important in a globalized health care environment where clinicians routinely serve communities whose traditions differ from their own. Practical bedside strategies include

- asking patients about cultural or ancestral practices related to illness or wellness.
- inviting family, community, or spiritual caregivers into care when desired.
- integrating movement or nourishment strategies safely alongside treatment.
- allowing space for meaning-making, ritual, or awe within care plans.

**Why nurses need this framework now**. Integrating cultural humility has been shown to increase satisfaction, strengthen therapeutic alliance, and improve engagement with care.^2^ When patients feel seen, not only as individuals but also as descendants, they are more likely to trust, return, participate, and heal. The Health Immemorial Framework aligns with this trajectory. It asks nurses to care not only for the illness before them but also for the stories behind it. The future of nursing requires honoring the past. Culture is not an accessory to care; it is the vessel through which health is lived, felt, and inherited.

Health is not new. It is immemorial.
